# Spectral CT in practice: insights from an International Atomic Energy Agency survey

**DOI:** 10.1186/s13244-025-02109-z

**Published:** 2025-10-16

**Authors:** Virginia Tsapaki, Zoe Brady, Nathaly Barbosa, Marc Kachelrieß, Mahadevappa Mahesh, Egor Titovich, Bronwin Van-Wyk, Mauro Carrara

**Affiliations:** 1 Dosimetry and Medical Radiation Physics Section, Human Health Division, Department of Nuclear Science and Applications, International Atomic Energy Agency, Vienna, Austria; 2 Department of Radiology and Nuclear Medicine, Alfred Health, Melbourne, VIC Australia; 3 Department of Neurosciences, Central Clinical School, Monash University, Melbourne, VIC Australia; 4 Centre for Epidemiology and Biostatistics, School of Population and Global Health, University of Melbourne, Melbourne, VIC Australia; 5 Imaging and Nuclear Medicine Unit, Cancer Treatment and Research Center (CTIC), Bogota, Colombia; 6 Division of X-Ray Imaging and Computed Tomography, German Cancer Research Center, Heidelberg, Germany; 7 The Russell H. Morgan Department of Radiology and Radiological Science, Johns Hopkins University School of Medicine, Baltimore, MD USA; 8 Department of Medical Physics, Dr George Mukhari Academic Hospital, Gauteng Province, South Africa

**Keywords:** CT, Spectral, Clinical use, Quality assurance

## Abstract

**Introduction:**

Spectral CT is an advanced imaging technique that distinguishes X-ray energies at the detector level to provide energy-resolved images for enhanced tissue characterization and quantitative capabilities. Despite its increasing availability, there is limited information on global clinical use and quality assurance. To address this gap, the International Atomic Energy Agency conducted a global survey to assess current practices and inform future guidance development.

**Methods:**

An online survey was disseminated internationally between October 2024 and June 2025. The questionnaire collected data on the presence and use of spectral CT, quality assurance (QA) and quality control (QC), clinical applications, dosimetry, and training. Responses were analyzed using descriptive statistics.

**Results:**

Eighty-three validated responses were received. Only a small fraction of spectral CT systems was photon-counting, nearly all installed from 2024 onward. A wide range of clinical applications was reported, including oncology, urology, cardiology, and neurological imaging. While many institutions reported access to spectral CT, utilization remains limited. Many facilities do not perform dedicated quality control (53%) and retain dosimetry practices (61%). Training gaps—especially among medical physicists—were evident. Respondents cited barriers such as insufficient training, workflow complexity, additional time for image review, limited post-processing tools, and uncertainty about clinical relevance.

**Conclusions:**

This preliminary global survey reveals that while spectral CT access is expanding, its clinical integration and QA/QC practices remain inconsistent. Standardized protocols, targeted training—particularly for medical physicists—and international guidance are urgently needed to support safe and effective adoption of spectral imaging in routine clinical care.

**Critical relevance statement:**

This global IAEA survey highlights critical gaps in spectral CT implementation and quality assurance, providing foundational insights that support future clinical advances through standardized protocols, targeted training, and globally harmonized guidance for more effective integration into radiology practice.

**Key Points:**

- Spectral CT adoption outpaces its clinical use and quality assurance integration.
- The survey shows limited training, quality control, and routine spectral CT use.
- Standardized guidelines and targeted training are essential for broader implementation.

**Graphical Abstract:**

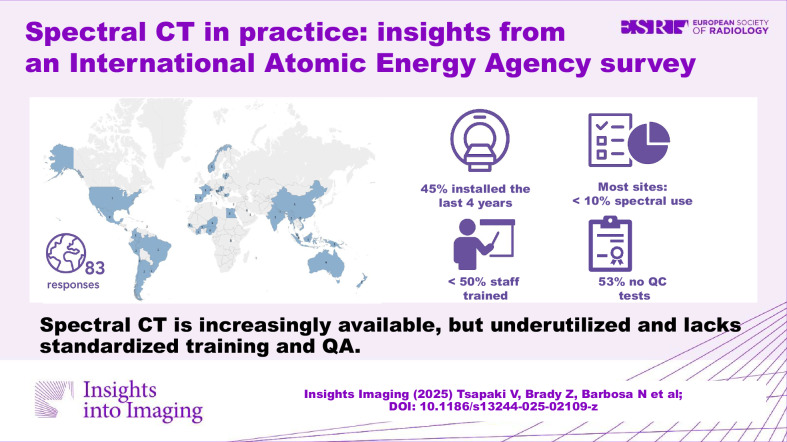

## Introduction

Computed tomography (CT) has emerged as a fundamental modality in diagnostic radiology, owing to its ability to acquire volumetric datasets with high spatial and temporal resolution in a short acquisition time [1, 2]. It plays a pivotal role in clinical decision-making by providing comprehensive diagnostic information across multiple phases of care, including initial diagnosis, disease staging, treatment planning, response assessment, and follow-up surveillance [3]. With the global population aging and the incidence of chronic conditions such as cardiovascular disease, cancer, and neurodegenerative disorders on the rise, the demand for advanced imaging continues to grow [4].

This increasing clinical reliance has been accompanied by rapid technological evolution. CT detectors have progressed from single-row to multi-row arrays, and now to energy-resolving spectral detectors that enable energy-selective imaging. These newer technologies enhance diagnostic accuracy by improving lesion conspicuity, reducing artifacts, and enabling quantitative assessments [5–11]. In particular, the advent of spectral CT has introduced a paradigm shift by enabling energy-resolved imaging, which allows for the differentiation of tissues based on their atomic number and effective Z-material decomposition [12, 13]. The most advanced spectral CT with true energy discrimination is achieved with photon-counting CT systems. However, in the clinical setting, CT scanners with dual energy capabilities are becoming more available. These systems produce datasets from two different energy spectra through a variety of vendor-specific hardware options, including two X-ray tubes, fast-kilovoltage switching, dual-layer detector arrays or a simpler split filter method [14–16].

Simultaneously, innovations in iterative and artificial intelligence-driven image reconstruction, tube current modulation, automatic exposure control, and personalized dose optimization have significantly improved image quality while reducing patient radiation exposure [17, 18]. However, CT remains one of the largest contributors to medical radiation dose worldwide, with the main reason being that the volume of CT scans being performed continues to increase globally [19, 20]. The balance between diagnostic benefit and radiation risk necessitates strict adherence to the principles of justification and optimization [21]. Therefore, a strong quality assurance (QA) framework is vital—not only to uphold image quality and safety but also to adapt to the increasing complexity and sophistication of CT technology.

In response to these challenges and opportunities, the International Atomic Energy Agency (IAEA) emphasizes a comprehensive and systematic approach to QA in diagnostic imaging [22]. The current IAEA guidance document on QA in CT was published over a decade ago and does not adequately reflect the significant technological advancements that have since transformed CT practice [23]. To address this gap and inform the development of updated QA guidelines that encompass these innovations, a global survey was conducted to assess the current status of spectral CT use and QA across a range of clinical settings and geographical regions. This paper presents a detailed analysis of the survey results, offering insight into existing practices, challenges, and areas in need of harmonization or improvement.

## Methods

The survey aimed to gather comprehensive data on the current use, clinical applications, and QA practices related to spectral CT systems. Data collection for the survey was conducted using the International Research Integration System, an online platform developed and maintained by the IAEA [24]. The platform enables secure collection, storage, and integration of diverse data types—including structured numerical data, unstructured text responses, or images with built-in capabilities for automated metadata and tag extraction from medical images.

The survey was broadly disseminated to medical physicists, radiologists and medical radiation technologists through multiple channels, including direct emails, professional organizations, as well as social media, to ensure maximum outreach. This approach was designed to avoid any bias based on size or institutional profile, allowing all institutions an equal opportunity to participate. Data was collected between October 2024 and June 2025 from participating institutions worldwide and included facility identifiers and location (institution name, city, country), contact details, and basic country-level context. It then recorded CT inventory and spectral capability, followed by detailed QA information, dosimetry practice and technical protocol use. Clinical application and utilization were explored via questions on the indications for spectral CT and an open-ended item asking why spectral CT is not regularly used where applicable. Finally, the survey asked about human resources and education. Responses comprised a mixture of categorical items, numeric counts and free-text fields and were exported for descriptive analysis and validation, primarily using Microsoft Excel for data handling, visualization, and trend evaluation.

## Results

**Fig. 1:**
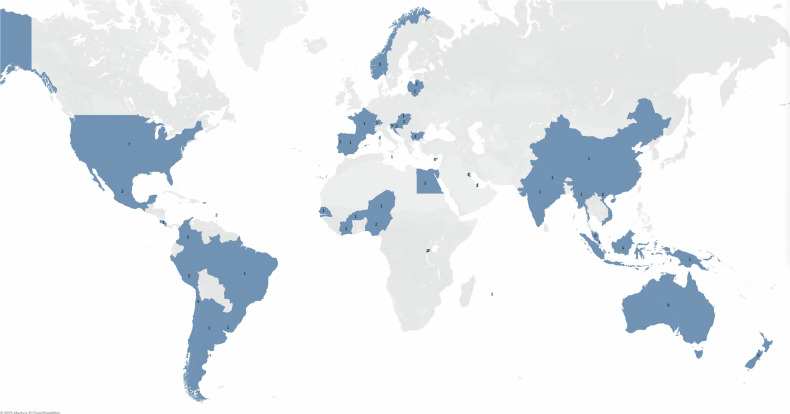
Geographic distribution of responses

A total of 91 responses were received through the spectral CT survey. Following data validation, 8 submissions were deemed invalid due to duplication, incomplete or inconsistent information, resulting in 83 responses being included in the final analysis. Figure 1 presents the geographic distribution of responses. Most responses came from the Asia-Pacific (27) and Europe (25), highlighting significant engagement from these two regions. Latin America also contributed a notable number of responses (14), followed by Africa with 10 and the United States of America (USA) with 7. Overall, the distribution shows a strong response from regions outside North America, particularly from Asia-Pacific and Europe. Specifically for Africa, it is noteworthy that responses were received from countries that are not traditionally among the largest or most resourced in terms of medical technology infrastructure; notably, countries like Niger, Rwanda, and Senegal, indicating interest in advanced CT technology even in settings not traditionally seen as high resource. However, several of these nations have demonstrated strong commitments to healthcare development and reform, which may explain their engagement with this technology. This is an encouraging sign of increasing awareness and prioritization of diagnostic imaging capabilities, even in settings with limited resources.

**Fig. 2:**
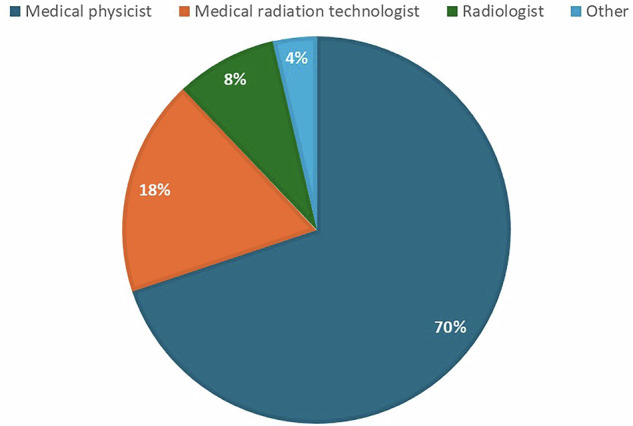
Distribution of responses based on professional category

Figure 2 shows the distribution of respondents by professional role. The majority (70%) identified as medical physicists, reflecting the dominant representation of this group in responses. Medical radiation technologists accounted for 18%, while radiologists made up 8%. A small portion (4%) selected Other, indicating limited participation from professions outside the core imaging specialties. The high proportion of medical physicist respondents reflects the survey’s primary focus on the technical aspects of spectral CT and its design, which did not primarily aim to assess clinical decision-making or diagnostic interpretation.

**Fig. 3:**
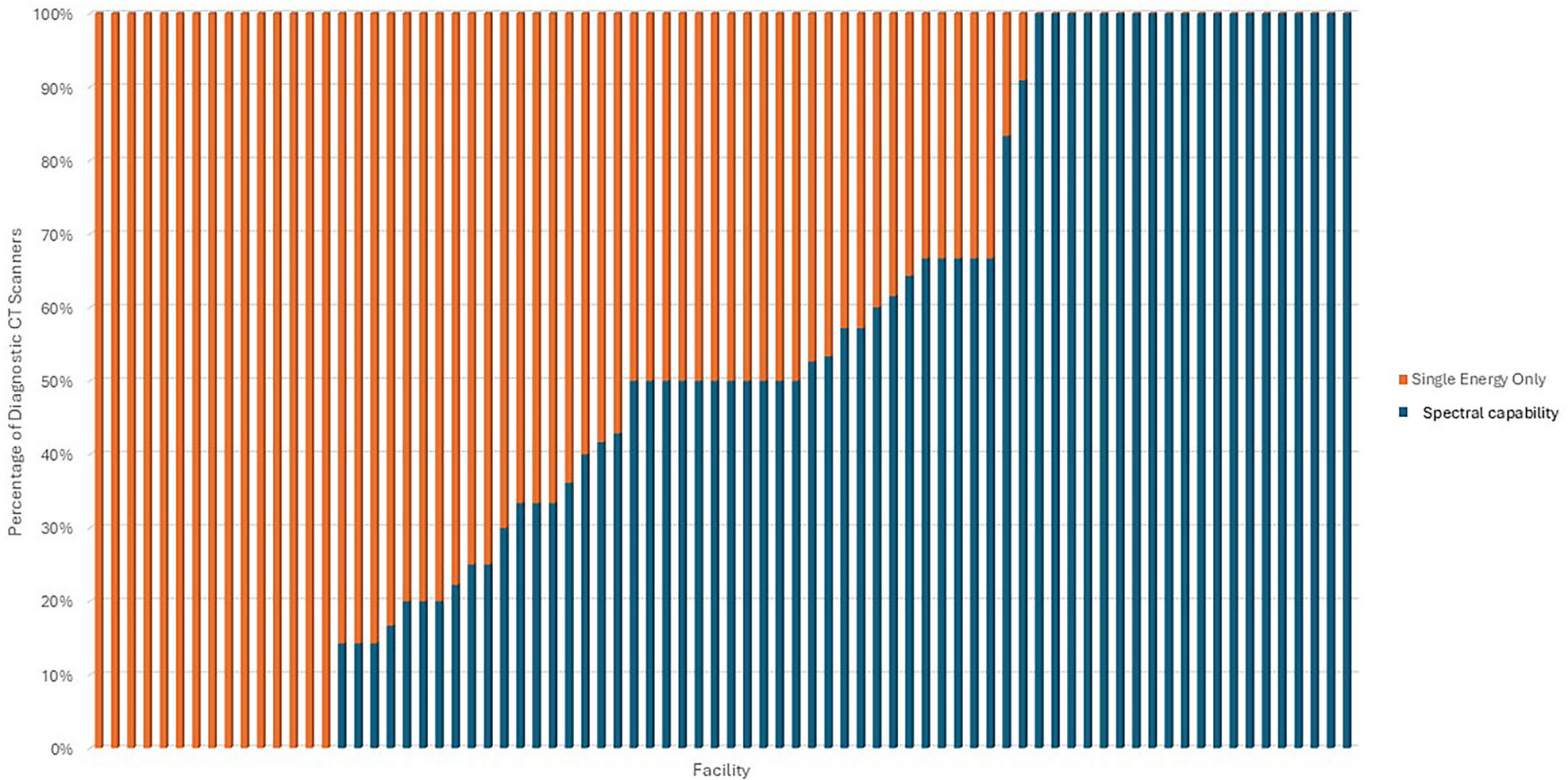
Percentage of diagnostic CT scanners in each facility with spectral capability

Figure 3 illustrates the percentage of diagnostic CT scanners in each facility that are equipped with spectral capability. Facilities are arranged in ascending order of spectral-equipped scanner percentage. The chart shows a wide variation across facilities, with many having either no spectral-capable scanners or a full complement of them. Certain facilities operate exclusively with single-energy scanners (orange), while others operate entirely with spectral-capable systems (blue). The figure highlights the growing presence of spectral capability, especially evident in the large number of facilities where 100% of scanners are spectral-capable.

**Fig. 4:**
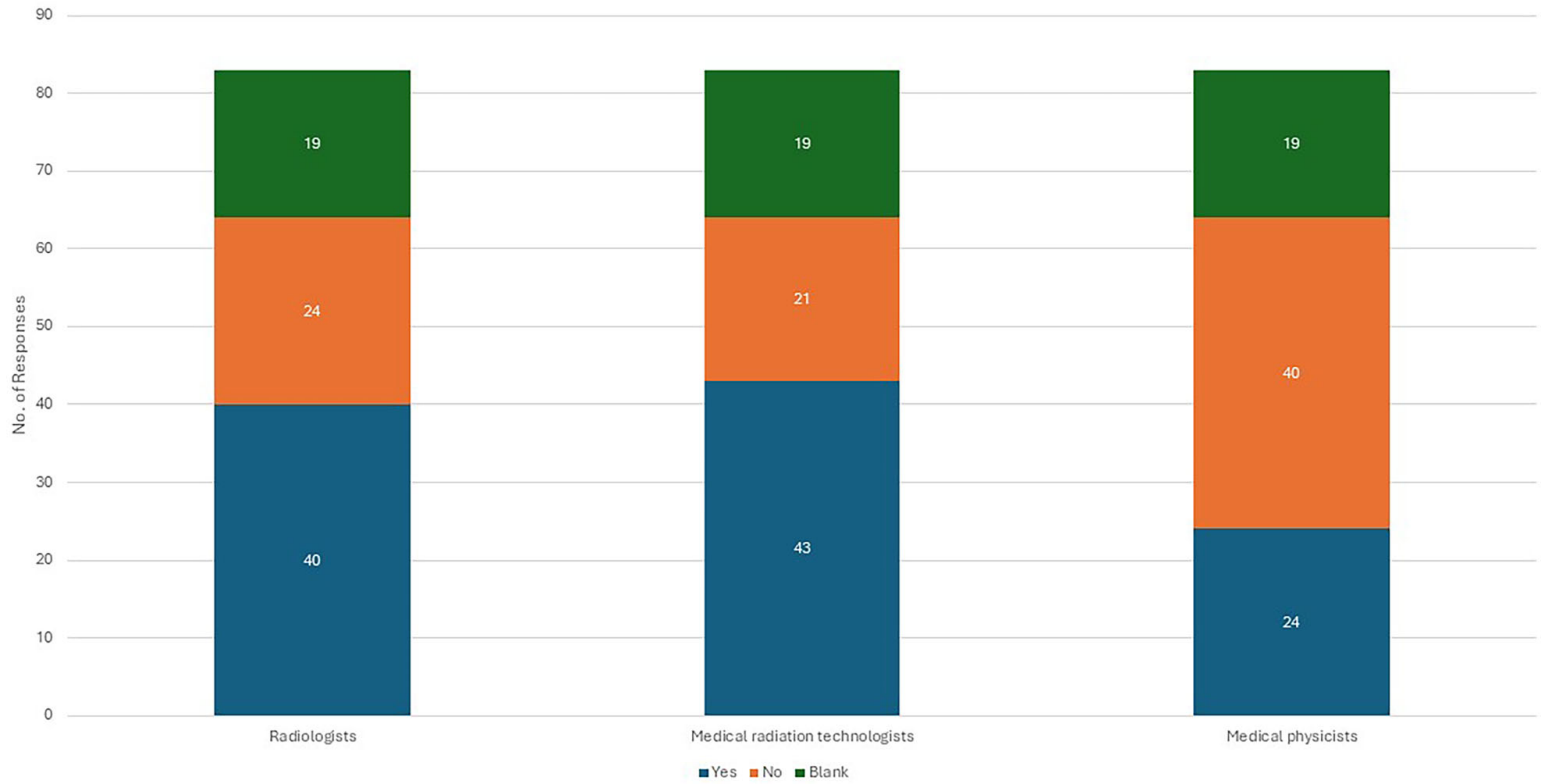
Distribution of responses related to staff-specific training in spectral CT, categorized by professional role

Figure 4 illustrates the breakdown of responses regarding whether staff received specific training in spectral CT, categorized by professional role. In 40 of the responding institutions, radiologists had received some spectral CT training, whereas in 24 institutions, radiologists received no training at all, and 19 did not provide any response. A similar pattern was observed for medical radiation technologists: 43 institutions reported that medical radiation technologists were trained, 21 reported they were not trained, and 19 institutions did not provide a response. In contrast, medical physicists showed a lower number of trained individuals (24), with 40 reporting no training and 19 not responding. These results suggest that while a portion of staff across all groups have received some form of training, there is still a significant gap—especially among medical physicists—where the majority report a lack of specific training in spectral CT.

Regarding the distribution of CT scanner manufacturers reported by respondents, Siemens systems account for 62%, GE for 21%, Canon for 10%, and Philips for 7%. While the overall response rate to the survey was relatively modest, the results reflect a range of CT technologies in clinical use across the responding sites, highlighting the diversity of systems represented in the sample. The installation years for the CT systems reported span a 16-year period, ranging from 2009 to 2025. This distribution indicates a significant recent investment in CT technology, with nearly half (45%) of the systems installed in just the past 4 years. While 55% were installed between 2009 and 2021, the surge in more recent installations suggests a trend toward modernization and likely increased availability of advanced features such as spectral imaging in newer systems. Of 168 spectral CT systems listed by respondents, eleven were photon-counting CT scanners. The vast majority of these (9 of 11) have been installed from 2024 onward.

**Fig. 5:**
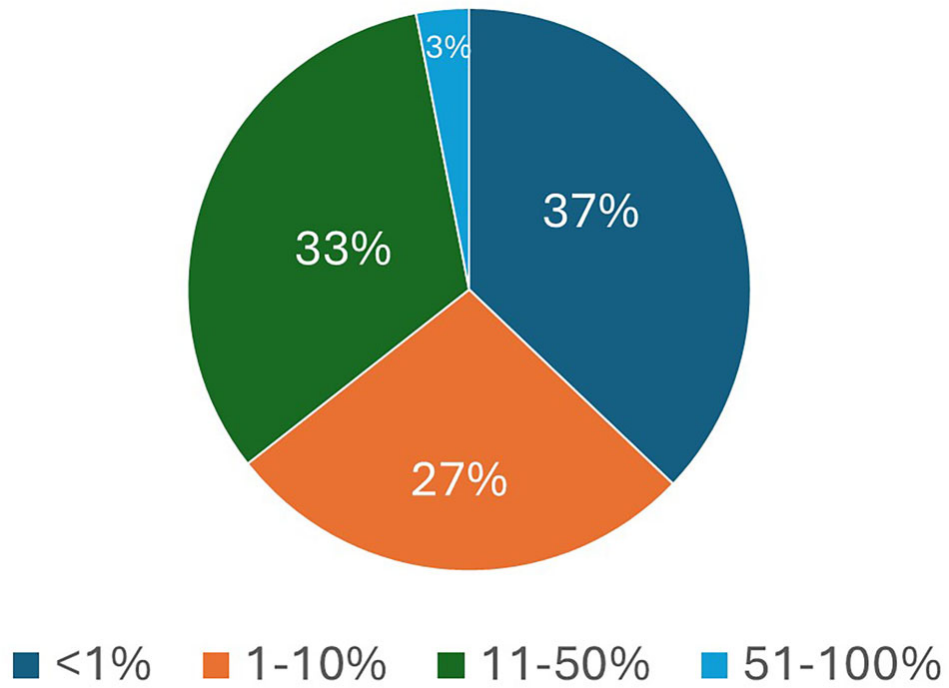
Weekly CT examination proportion of spectral scans per scanner expressed in percentages

Figure 5 illustrates the proportion of weekly CT examinations performed using spectral imaging across surveyed scanners. Most CT scanners use this technology for a small fraction of cases: 37% of scanners perform spectral imaging in less than 1% of weekly examinations, and 27% use it in less than 10% of cases. Thirty-three percent (33%) reported spectral use in 10–50% of their scans, while only 3% of scanners perform spectral imaging in more than half of their weekly examinations. This distribution suggests that while spectral capability may be present, its routine clinical use remains limited in many facilities. One reason may be that most spectral scans require dedicated scan protocols to be selected prospectively, i.e., before the scan starts.

Most respondents (53%) indicated that specific QC tests for spectral CT are not performed, suggesting that dedicated protocols are not yet widely implemented. Only 19% reported having such tests in place, while another 19% did not respond—possibly due to uncertainty or lack of relevance. This points to a general trend of treating spectral CT like conventional CT, with limited use of specialized QA procedures. Similarly, 61% of respondents stated that dosimetry practices remain unchanged for spectral CT, and only 18% reported using different dose assessment methods. The remaining 21% did not answer, indicating possible gaps in awareness or experience. These findings highlight the need for clearer guidance and standardization in both QA and dosimetry practices for spectral CT.

As far as the frequency of QC testing is concerned, responses reveal a wide variation in the types and frequency of these tests performed for spectral CT systems. Some facilities perform daily or weekly system calibrations, warm-up routines, and tube checks, while others conduct periodic manufacturer-recommended constancy tests using phantoms such as those supplied by the vendor, Catphan® (phantomlab, USA), CIRS (Sun Nuclear, Mirion Medi-cal, USA), or the American College of Radiology Gammex 464 CT phantom (Sun Nuclear, USA). Common QC elements include assessments of image quality, slice thickness, noise, uniformity, spatial resolution and CT dose index. Some sites also use automated vendor tools for consistency checks. Overall, while some facilities have implemented regular QC protocols tailored to spectral imaging, others appear to rely on more general or minimal approaches.

Another question of the survey asked respondents to describe the clinical applications for which they use spectral CT. The responses reveal a wide array of uses across multiple specialties and clinical scenarios, highlighting the versatility of spectral imaging. Pulmonary embolism emerged as one of the most common applications, especially in older patients or those requiring reduced contrast due to renal impairment. Abdominal imaging—including liver, pancreas, kidney, and gastrointestinal evaluations—was frequently mentioned, often for lesion characterization, perfusion analysis, or low-contrast-dose studies. Urological applications such as kidney stone detection and uric acid versus calcium differentiation were also prevalent. Several sites reported use in cardiac or oncologic imaging, musculoskeletal assessments, and routine radiation therapy planning—particularly for head, neck, prostate, and spine. Cardiac applications included coronary CT angiography, transcatheter aortic valve replacement planning, congenital heart disease, and myocardial infarct visualization. Neurological uses ranged from stroke assessment and post-operative brain scans to differentiating hemorrhage from contrast. Spectral CT was also used for virtual non-contrast imaging, metal artifact reduction, iodine mapping, and interventional planning. Some respondents indicated routine spectral CT use across nearly all protocols, while others noted limited or discontinued use, either due to technical constraints, software limitations, or lack of clinical demand. A few also mentioned that spectral CT data are acquired automatically, but not always analyzed or reported.

The last free-text question asked respondents to explain why spectral CT is not regularly utilized at their facility. The responses reveal a wide range of reasons, highlighting both technical and operational barriers to routine use. These could be categorized into 5 major categories:

- Knowledge and training barriers: A major theme was a lack of understanding or training on how to interpret or apply the additional information provided by spectral CT imaging in clinical practice.
- Clinical relevance and demand: Several respondents noted that while it provides extra data (such as perfusion maps or virtual non-contrast images), this information is not always considered clinically useful or relevant, leading to limited utilization. Low clinical demand, limited indications, and lack of physician requests were common reasons for limited use. In some settings, spectral CT was only employed for very specific applications, such as cardiac imaging or oncology, while other departments relied entirely on conventional single-energy CT.
- Technical and workflow limitations: Technical limitations were also frequently mentioned, such as a lack of post-processing software or incompatibility with newer tools that manufacturers offer. Some facilities noted that spectral protocols are only available on specific scanners or require separate acquisition modes, making them less practical for routine workflows. Additional time for image processing and review, along with concerns about increased radiation dose, were also noted as deterrents.
- Operational and administrative barriers: A few responses cited administrative or vendor-related barriers, including software being locked or deemed not valuable by hospital managers.
- Variation in use and access: Despite these challenges, some respondents reported frequent or routine use of spectral CT, suggesting that its adoption and perceived value vary widely depending on clinical setting, equipment availability, and staff familiarity.

## Discussion

To our knowledge, this is the first international survey on a global scale and provides valuable insights into the current status of spectral CT technology implementation, clinical use, and QA practices across diverse healthcare settings. The findings highlight a mixture of progress, variability, and persistent barriers to the broader adoption of this advanced imaging technology.

Training gaps emerged as a recurrent theme. Although radiologists and medical radiation technologists reported relatively balanced exposure to spectral CT training, medical physicists -despite their dominant representation in the sample- had the highest proportion of untrained respondents. This suggests a critical need for targeted educational initiatives across all professional roles with a strong focus on medical physicists, particularly as spectral imaging becomes more integrated into clinical protocols. Despite the widespread availability of spectral CT, the implementation of tailored QA protocols remains inconsistent. The findings underscore the need for professional guidelines and training resources to support QA in spectral imaging. Currently, the only professional guidance available for QC in spectral CT is the American Association of Physicists in Medicine Task Group Report 299 [25].

While spectral CT is used in a wide array of applications in participating institutions—including cardiology, abdominal and pancreatic imaging—the frequency of use remains low in most facilities. Over 60% of scanners perform spectral imaging in less than 10% of weekly exams. This mismatch between availability and utilization reflects a broader uncertainty around clinical relevance, value-added interpretation, and integration into routine workflow. Free-text responses highlighted several barriers, including a lack of awareness of spectral data utility, limited radiologist familiarity, concerns about additional radiation dose, and software or post-processing limitations. Only two respondents in the survey mentioned additional radiation dose as a barrier to the use of spectral CT, indicating that this is not a widespread concern among users. Several respondents indicated that spectral CT demands more time for reconstruction, review, and interpretation than conventional CT, partly because it produces a greater number of images, posing challenges in high-throughput clinical environments. High patient volumes and limited staff time further compound this issue, making it difficult to incorporate spectral post-processing into routine workflows. As a result, even when the technology is available, practical constraints often lead to limited utilization. These findings reinforce the notion that technological capability alone is insufficient without corresponding investment in workflow integration, clinical education, and user confidence. Interestingly, none of the respondents explicitly cited funding or reimbursement as a barrier to the use of spectral CT. While economic considerations are known to influence medical imaging use in many healthcare systems, particularly those driven by reimbursement incentives, this issue did not emerge in the survey responses. This may reflect the technical focus of the survey and the professional background of the respondents, or regional differences in how medical imaging services are funded.

The reasons cited for limited or non-use of spectral CT vary across practical, technical, and organizational domains. Beyond training and software gaps, many respondents reported low clinical demand or limited indications within their practice. In some cases, hospital administrators deemed spectral functionality non-essential, even locking software features due to perceived low value. Yet, others indicated routine use and broad adoption, highlighting that institutional culture, leadership, and user engagement can drive successful implementation. This variability underscores the importance of context-specific strategies to support adoption—such as tailored clinical guidelines, case-based learning, and evidence generation through multicenter studies.

Our findings align with observations from the literature regarding the practical and clinical challenges associated with advanced CT technologies [15]. Spectral CT generates a larger volume of data compared to conventional CT, requiring additional post-processing time and greater computational resources [26]—a concern echoed by multiple respondents in our survey. Although not explicitly mentioned, a common limitation across many vendors is the requirement to decide prospectively—before image acquisition—whether to use spectral imaging. This pre-scan decision adds complexity to the workflow and may discourage routine use, especially in high-throughput settings [27]. Developments in automation, such as those powered by artificial intelligence, could potentially offer opportunities to streamline data selection, preprocessing, and visualization, reducing the interpretative burden on clinicians [28]. For spectral CT to be widely adopted in routine practice, acquisition must become more seamless and interpretation more intuitive. Many participants highlighted that time constraints and high patient throughput limit their ability to fully utilize spectral imaging, even when technically available. Furthermore, as noted in recent studies, while advanced CT modalities can produce high-quality, customizable images that may enhance diagnostic confidence, their actual impact on clinical decision-making remains uncertain. This uncertainty, reflected in our findings, contributes to hesitation or selective use of spectral CT, especially when the added complexity is not clearly tied to improved patient outcomes.

Our results also echo concerns raised in the literature about the variability introduced by differences in hardware and post-processing algorithms across CT vendors [26, 29–31]. Although our survey did not focus on detailed technical comparisons between manufacturers, the wide variation in spectral CT implementation reported suggests that cross-vendor variability may play a role in limiting standardization and reproducibility, particularly important in oncological imaging [26]. Until consensus guidelines are established, the clinical adoption of spectral CT may remain fragmented and highly dependent on local protocols and vendor-specific capabilities.

The survey also had some limitations. The geographic distribution was skewed toward Asia-Pacific, Latin America and Europe, with modest representation from the USA and the Middle East. Moreover, spectral CT utilization was captured as broad weekly proportions rather than at the exam or indication level, precluding stratified analyses by clinical scenario, patient subgroup, or protocol type and constraining exploration of how utilization varies with specific QA/QC practices. Greater regional balance and exam-level granularity in the future can enable more robust comparisons and clearer linkage between use patterns and quality processes.

## Conclusion

This IAEA global survey is the first international effort to assess current practices in spectral CT and guide future studies. The findings show that although access to spectral CT is expanding—even in resource-limited settings—its routine clinical use remains limited. Major barriers include a lack of standardized QA protocols, insufficient training, and workflow challenges, despite the availability of systems from all major vendors. Successful integration requires more than just access to technology; it depends on clear clinical protocols, targeted training—particularly for medical physicists—and greater awareness of spectral CT’s clinical value. Broader adoption will require coordinated efforts among manufacturers, professional societies, and healthcare institutions to develop harmonized, evidence-based guidelines and training resources.

## Data Availability

The datasets generated and analyzed during the current study are available in an Excel file and can be shared with the journal editor or reviewers if needed.

## Abbreviations

CT: Computed tomography
IAEA: International Atomic Energy Agency
QA: Quality assurance
QC: Quality control
